# Navigating pain and appetite challenges in palliative care for pancreatic cancer: insights from a national, longitudinal consecutive cohort study

**DOI:** 10.1007/s00520-025-09402-z

**Published:** 2025-04-08

**Authors:** Mariana S. Sousa, Maja Villanueva Garcia, Megan Blanchard, Barbara Daveson, David Currow, Nadia N. Khan, David Goldstein, Merran Findlay, Amanda Landers, Meera R. Agar

**Affiliations:** 1 Improving Palliative, Aged and Chronic Care Through Clinical Research and Translation (IMPACCT), Faculty of Health, University of Technology Sydney, PO Box 123Broadway , Sydney, NSW 2007 Australia; 2 School of Nursing and Midwifery, Western Sydney University, Sydney, NSW Australia; 3 Palliative Care Outcomes Collaboration (PCOC), Faculty of Science, Medicine and Health, University of Wollongong, Wollongong, NSW Australia; 4 Graduate School of Medicine, Faculty of Science, Medicine and Health, University of Wollongong, Wollongong, NSW Australia; 5 Wolfson Palliative Care Research Centre, University of Hull, Hull, England; 6 Cancer Research Program, School of Public Health and Preventive Medicine, Monash University, Melbourne, VIC Australia; 7 Prince of Wales Clinical School, University of NSW, Sydney, NSW Australia; 8 Maridulu Budyari Gumal (SPHERE) Cancer Clinical Academic Group, University of NSW, Sydney, NSW Australia; 9 Cancer Services, Royal Prince Alfred Hospital, Sydney Local Health District, Sydney, NSW Australia; 10 Chris O’Brien Lifehouse, Sydney, NSW Australia; 11 Cancer Care Research Unit - The Daffodil Centre, a Joint Venture with Cancer Council, University of Sydney, Sydney, NSW Australia; 12 Department of Medicine, University of Otago, Christchurch, New Zealand; 13 South Western Sydney Clinical School, University of New South Wales Sydney, Sydney, NSW Australia

**Keywords:** Pancreatic cancer, Palliative care, Distress, Appetite, Pain, Symptom burden

## Abstract

**Purpose:**

Pancreatic cancer poses significant challenges in symptom management. Many people have intractable pain and anorexia which is often not amenable to current available options for palliation. This study aims to outline the longitudinal patterns and assess the burden of distress related to pain and appetite experienced by individuals with pancreatic cancer in people referred to Australian palliative care services.

**Methods:**

Consecutive national cohort study using point-of-care data on symptom distress in people referred to specialist palliative care services.

**Results:**

From 2013 to 2022, information from 20,558 care episodes involving 15,536 people with pancreatic cancer referred to 203 palliative care services nationally were included. Similar numbers of people were admitted to inpatient and community services, with 69% and 60% reporting distress due to pain and appetite, respectively. Distress extended to sleeping (79%, 82%), nausea (83%, 85%), bowels (80%, 83%), breathing (70%, 77%) and fatigue (77%, 77%) for pain and appetite, respectively. Strongest associations were with psychological/spiritual issues (inpatient OR 1.78, 95% CI 1.66–1.90) and pain severity (community OR 1.51, 95% CI 1.42–1.60) for appetite-related distress and pain severity (inpatient OR 1.60, 95% CI 1.49–1.72; community OR 1.35, 95% CI 1.27–1.44) for pain distress. Trends within the cohort revealed increased mild appetite-related distress (+ 10%) and decreased moderate (- 22%) and severe (- 11%) distress, with similar trends for pain.

**Conclusion:**

This study underscores prevalent distress in people with pancreatic cancer in Australian palliative care, highlighting the need for optimised referrals and strategies targeting pain severity, appetite-related concerns and psychological and spiritual aspects for improved care outcomes.

## Introduction

### Background

Pancreatic cancer ranks as the eighth most commonly diagnosed cancer in Australia, and it is highly fatal, with an estimated mortality rate of 80%, placing it as the fourth leading cause of cancer death [1]. Despite incremental improvements in survival rates, it maintains one of the lowest 5-year survival rates at less than 11%, in stark contrast to the 70% average for all cancers combined [2, 3]. Among candidates eligible for surgery, those undergoing resection and adjuvant therapy still face only a median survival of 20–28 months [4].

Pancreatic cancer is also one of the most complex cancers in terms of adequate symptom management, with pain and anorexia highly prevalent in this group. Approximately 50% of patients present with pain at the time of diagnosis, with 70% experiencing appetite issues [5]. Symptoms of pain and anorexia are often interlinked with each other in this setting and contribute to significant psychological impacts on patients and their caregivers [6], highlighting the pressing demand for more effective interventions.

There is evidence to suggest that supporting interventions such as pain management, nutritional interventions and food advice offer avenues to enhance symptom control [6]. Such interventions are embedded within palliative care, and as such, palliative care can play a critical role in the management of patients with pancreatic cancer. Engagement with these services has been associated with clinically significant improvements in quality-of-life and symptom burden [7] as well as demonstrating potential to reduce emergency department admissions [8] and reduce (perceived) inappropriate end-of-life care [9–12].

### Rationale

Optimal care pathways for pancreatic cancer have been developed in Australia [13] and a National Pancreatic Cancer Roadmap has identified symptom management as a priority [14]. Despite the need for symptom control and the identified benefits from linking people with pancreatic cancer to palliative care services, few data exist on the pain and appetite needs of people with pancreatic cancer. In the Australian context, our study seeks to explore pain and appetite problems in a consecutive cohort of people with pancreatic cancer referred to Australian palliative care services, contributing insights to unmet needs. The significance of looking at these data is to further our knowledge about the prevalence of distress related to pain and appetite in pancreatic cancer, so we can offer the most appropriate support when needed, irrespective of the place where care is being provided.

### Objectives

To explore the patterns and burdens (symptom-related distress and symptom- and problem-related severity) of pain and appetite experienced by people with pancreatic cancer referred to palliative care services in Australia, including any changes in referral patterns to Australian palliative care services over time.

## Methods

### Study design and setting

This is a consecutive, longitudinal cohort study of prospectively collected point-of-care patient information captured by the Australian national Palliative Care Outcomes Collaboration (PCOC) quality improvement program. To this end, Australia-wide palliative care services voluntarily participate and collect information on all patients at agreed transition points in care. PCOC consists of standardised, clinical assessment tools used to drive improvements in palliative care outcomes, through monitoring patient outcomes at a service level and benchmarking national services [15]. Access to specialist palliative care services in Australia is referral-based and includes community services (i.e. outpatient clinics, community care including care in residential aged care facilities) and inpatient hospital services (i.e. direct care, consultative care, designated palliative care bed and standalone services). This study is reported in accordance with The Strengthening the Reporting of Observational Studies in Epidemiology (STROBE) Statement: guidelines for reporting observational studies [16].

### Population

Inclusion criteria included all patients aged ≥ 18 years who had a diagnosis of pancreatic cancer and were in the care of one of the 203 Australian specialist palliative care services registered with the PCOC program between 01/01/2013 and 31/12/2022. Only patients with at least one recorded measurement of pain- and/or appetite-related distress at the first assessment were included from the PCOC dataset.

### Data sources

Patient-level data were collected at the start of an episode of care (a new place or setting of care) which is defined as a continuous period of care for a patient (or resident) in one setting. An episode of care can occur at any period between referral to palliative care and last contact with palliative care. Patients were followed across service settings using a unique service-based identifier based on name, birth dates and sex. People referred to palliative care, often experience multiple episodes over time across different care settings (e.g. hospital, home-based, ambulatory). Patient-level data included demographic characteristics (sex, age group, country of birth and preferred language spoken at home) and geographical location. Assessments data were collected at the commencement of each care episode, at the point of transition between each clinically important change in condition (i.e. stable, unstable, deteriorating or terminal phases) during the episode [17] and, for episodes not ending in death, at the final available assessment prior to discharge from service (or change in setting of care).

### Measures

Symptom-related distress was measured using the PCOC Symptom Assessment Scale (SAS). This is a patient (or proxy, if the patient cannot self-report) rated tool that measures the level of distress caused by seven symptoms in palliative care (i.e. pain, appetite problems, nausea, bowel problems, breathing problems, difficulty sleeping and fatigue) [18, 19]. Using the PCOC SAS, patients are asked to rate their distress on a 0–10 numerical rating scale (NRS). A score of 0 denotes that the symptom is absent, 1–3 is mild, 4–7 is moderate and 8–10 is severe symptom distress [19].

The Palliative Care Problem Severity Scale (PCPSS) measures the severity of symptoms and is completed by clinicians [20]. The following domains are assessed by the PCPSS: pain, psychological/spiritual, other symptoms and family/carer. Each domain is rated on a 4-point scale, with 0 denoting that the symptom is absent, 1 is mild, 2 is moderate and 3 is severe.

The level of performance of each patient was assessed with the Australia-Modified Karnofsky Performance Scale (AKPS) [21]. The AKPS was designed and validated to ensure application with palliative care patients across various care settings. Performance status is assessed on an ordinal scale from 0 (dead) to 100 (normal; no evidence of disease). AKPS scores provide valuable information about a patient’s ability to carry out daily activities and their prognosis, as well as assist in tacking changes overtime, helping the patient, healthcare providers and caregivers make informed decisions regarding care options.

### Statistical methods

Patient characteristics are described by sex, age group, preferred language spoken at home. For phase-level analyses (i.e. at the point of transition between each clinically important change in condition), the primary outcome was patient-reported pain- and/or appetite-related distress at the start and end of each palliative care phase. Descriptive statistics were also calculated for the incidence of moderate or severe pain- and/or appetite-related distress by care setting, patient characteristics and related measures.

Sub-group analyses were conducted by setting of care (community services and inpatient hospital services) to help understand how different environments influence patient outcomes, given at least one paper that suggests differential symptom control between the two settings [12]. Exploratory data analysis was performed on demographic characteristics, as well as level of performance (AKPS), level of symptom distress (PCOC SAS) and symptom or problem severity (PCPSS). This analysis helps to tailor care plans to address patients’ specific needs, such as the need for intensive symptom management or support for physical care. These insights can, in turn, inform decisions regarding transitions to different setting, such as moving from home to a hospital.

Separate logistic regressions for each setting were calculated to provide odds ratios (ORs) for the association between pain- and/or appetite-related distress (severe-moderate versus mild-absent) and distress related to cardinal symptoms. The odds ratio estimates were adjusted for sex, age group and AKPS at episode start. Confidence intervals for the odds ratios were estimated using a patient-clustered variance sandwich estimator to adjust for multiple assessments per patient. This was an exploratory analysis with no pre-planned hypotheses. All analyses were undertaken using SAS® v9.4M5 and R v3.6.2.

## Results

Data on a total of 15,536 patients with pancreatic cancer who presented to PCOC-registered palliative care services between January 1, 2013 and December 31, 2022 and provided at least one measure of pain- and/or appetite-related distress were included in the analysis. This involved 20,558 episodes of care and 37,185 assessments of clinically important changes in condition—phases (Table 1).

**Table 1:** Number of records included in the dataset, by episodes of care

	Total	1 episode^a^	2 episodes	3 + episodes
Patients	15,536	11,997	2559	660
Episodes	20,558	11,997	5118	3443
Assessments of clinically important changes in condition (phases)^b^	37,185	29,990	5220	1975
Episodes ending in death	7781	5913	1354	513
Median days between patient’s first and last assessment by the palliative care team (IQR)	5 (14)	5 (13)	6 (18)	7 (21)

^a^Episode refers to a continuous period of care for a patient (or resident) in one setting (i.e. hospital, private residence and residential aged care). The number of episodes is determined by the number of settings of care between referral to palliative care and last contact with palliative care

^b^Assessments data were collected at the commencement of each care episode, at the point of transition between each clinically important changes in condition (i.e., stable, unstable, deteriorating or terminal phases) during the episode

### Demographics and care setting

Table 2 presents an overview of patient characteristics and the care setting for both initial and subsequent episodes of care. The data indicate that sex was evenly distributed, and more were born in Australia (62%). The average age at death was 73.3 years (range 21–104). In terms of care setting, more patients initiated their first episode of care within the community, accounting for 55% of cases. This pattern persisted across all subsequent episodes of care.

**Table 2:** Demographic characteristics and utilisation by setting of care

		***N* (%)**
Sex	Female Male	7452 (48) 8084 (52)
Country of birth	Australia	9382 (62)
	Other country	5710 (38)
Preferred language	English	13,796 (90)
	Other language	1515 (10)
Age at death	Mean	73.3
	Median	74.0
	SD	11.8
	Minimum	21
	Maximum	104
Setting for first episode	Inpatient—designated palliative care bed	3496 (23)
	Inpatient—not designated palliative care bed^a^	1429 (9)
	Inpatient—not further specified	2019 (13)
	Community—private residence	1836 (12)
	Community—residential aged care	128 (0.8)
	Community—not further specified	6627 (43)
Setting for all^b^ episodes	Inpatient—designated palliative care bed	5116 (25)
	Inpatient—not designated palliative care bed^a^	1924 (9)
	Inpatient—not further specified	2366 (11)
	Community—private residence	2338 (11)
	Community—residential aged care	173 (0.8)
	Community—not further specified	8670 (42)

^a^Consult services seeing patients in non-specialist palliative care unit

^b^All episodes, including the first episode

### Clinical characteristics at first presentation

Table 3 presents the clinical characteristics and distress reporting of patients at their initial episode of care. Of the 15,536 patients, 10,705 (69%) reported distress due to pain and 9283 (60%) reported distress attributable to appetite issues.

**Table 3:** Clinical characteristics of people diagnosed with pancreatic cancer and seen by palliative care services in Australia upon admission, who reported distress due to appetite and pain

(*N*)		Inpatient *N* (%)				Community *N* (%)				Total *N* (%)			
		*No distress due to appetite*	*Distress due to appetite*	*No distress due to pain*	*Distress due to pain*	*No distress due to appetite*	*Distress due to appetite*	*No distress due to pain*	*Distress due to pain*	*No distress due to appetite*	*Distress due to appetite*	*No distress due to pain*	*Distress due to pain*
Sex	Male (8084)	1843 (50)	1821 (50)	1209 (33)	2455 (67)	1452 (33)	2964 (67)	1313 (30)	3103 (70)	3295 (41)	4785 (59)	2522 (31)	5558 (69)
	Female (7452)	1571 (48)	1706 (52)	1070 (33)	2207 (67)	1383 (33)	2792 (67)	1235 (30)	2940 (70)	2954 (40)	4498 (60)	2305 (31)	5147 (69)
Age in years at first episode start	<45 (220)	50 (51)	49 (49)	22 (22)	77 (78)	44 (36)	77 (64)	17 (14)	104 (86)	94 (43)	126 (57)	39 (18)	181 (82)
	45–54 (864)	201 (52)	187 (48)	83 (21)	305 (79)	167 (35)	309 (65)	77 (16)	399 (84)	368 (43)	496 (57)	160 (19)	704 (81)
	55–64 (2488)	547 (48)	590 (52)	282 (25)	855 (75)	446 (33)	905 (67)	302 (22)	1049 (78)	993 (40)	1495 (60)	584 (24)	1904 (76)
	65–74 (4446)	995 (50)	1013 (50)	611 (30)	1397 (70)	757 (31)	1681 (69)	668 (27)	1770 (73)	1752 (40)	2694 (60)	1279 (29)	3167 (71)
	75–84 (4814)	1006 (49)	1068 (51)	728 (35)	1346 (65)	920 (34)	1820 (66)	921 (34)	1819 (66)	1926 (40)	2888 (60)	1649 (34)	3165 (66)
	85+ (2704)	618 (50)	620 (50)	553 (45)	685 (55)	501 (34)	965 (66)	563 (38)	903 (62)	1119 (41)	1585 (59)	1116 (41)	1588 (59)
AKPS*	100-70 (3026)	261 (48)	279 (52)	145 (27)	395 (73)	950 (38)	1536 (62)	830 (33)	1656 (67)	1211 (40)	1815 (60)	975 (32)	2051 (68)
	50–60 (5027)	1035 (42)	1458 (58)	754 (30)	1739 (70)	1287 (30)	3047 (70)	1272 (30)	3062 (70)	2322 (34)	4505 (66)	2026 (30)	4801 (70)
	10–40 (5476)	2053 (55)	1709 (45)	1323 (35)	2439 (65)	584 (34)	1130 (66)	430 (25)	1284 (75)	2637 (48)	2839 (52)	1753 (32)	3723 (68)
PCOC SAS** (moderate/ severe scores)	Sleeping (2033)	198 (21)	729 (79)	146 (16)	781 (84)	226 (20)	880 (80)	214 (19)	892 (81)	424 (21)	1609 (79)	360 (18)	1673 (82)
	Nausea (2143)	227 (20)	886 (80)	185 (17)	928 (83)	142 (14)	888 (86)	145 (14)	885 (86)	369 (17)	1774 (83)	330 (15)	1813 (85)
	Bowels (2589)	286 (23)	980 (77)	236 (19)	1030 (81)	232 (18)	1091 (82)	211 (16)	1112 (84)	518 (20)	2071 (80)	447 (17)	2142 (83)
	Breathing (1458)	261 (33)	521 (67)	173 (22)	609 (78)	172 (25)	504 (75)	165 (24)	511 (76)	433 (29)	1025 (70)	338 (23)	1120 (77)
	Fatigue (6276)	661 (27)	1833 (73)	544 (22)	1950 (78)	778 (21)	3004 (79)	916 (24)	2866 (76)	1439 (23)	4837 (77)	1460 (23)	4816 (77)
PCPSS*** (moderate/ severe scores)	Pain (3584)	727 (39)	1140 (61)	74 (4)	1793 (96)	392 (23)	1325 (77)	34 (2)	1683 (98)	1119 (31)	2465 (69)	108 (3)	3476 (97)
	Other Symptoms (4283)	622 (32)	1351 (68)	472 (24)	1501 (76)	490 (21)	1820 (79)	491 (21)	1819 (79)	1112 (26)	3171 (74)	963 (23)	3320 (77)
	Psychological/ Spiritual (2440)	344 (33)	698 (67)	210 (20)	832 (80)	295 (21)	1103 (79)	280 (20)	1118 (80)	639 (26)	1801 (74)	490 (20)	1950 (80)
	Family/Carer (3272)	546 (40)	821 (60)	346 (25)	1021 (75)	456 (24)	1449 (76)	440 (23)	1465 (77)	1002 (31)	2270 (69)	786 (24)	2486 (76)

^a^AKPS: performance status is assessed on an ordinal scale from 0 (dead) to 100 (normal, no evidence of disease)

^b^SAS: A 10-point scale where a score of 0 denotes that the symptom is absent, 1–3 is mild, 4–7 is moderate and 8–10 is severe symptom distress

^c^PCPSS: a 4-point scale, with 0 denoting that the symptom is absent, 1 is mild, 2 is moderate and 3 is severe

### Age and distress

More than half of patients within each age group reported distress due to pain in both the inpatient and community settings. Across both settings, as the age group increased, the percentage of patients reporting distress from pain decreased.

### Performance status

Distress due to pain was reported by more than half of patients for each AKPS group in inpatient and community settings. Approximately half (52%) of patients in the 100–70 AKPS group and 58% of patients in the 50–60 AKPS group reported distress due to appetite problems in the inpatient setting. More than half of patients reported no distress due to appetite problems in the 10–40 AKPS group in the inpatient setting. A higher proportion of patients reported distress due to appetite problems in the community setting (62% of patients in the 100–70 group, 70% in the 50–60 group, 66% in 10–40 group).

### Symptom burden

The majority of patients experiencing distress related to symptoms other than pain and appetite reported it at moderate-to-severe levels in inpatient and community settings (Table 4). Nausea was the symptom with the highest proportion of all patients reporting distress due to pain or appetite problems. Overall, across both settings, caregivers also expressed concerns related to pain (76%) and appetite (69%) distress experienced by their loved ones.

### Severity of distress and associated symptoms

Most participants experiencing distress due to symptoms in the four PCPSS domains recorded moderate to severe scores for pain and appetite, across inpatient and community settings, as well as across the entire sample of the first episode (Table 3). Table 4 shows a detailed breakdown of severities of symptoms experienced by patients in this study. Most patients rated these symptoms as mild.

**Table 4:** Symptom severity and distress for the assessments of people with pancreatic cancer seen by palliative care services in Australia at first episode^a^

Symptom scale	Severity^b^	Inpatients (%)	Community (%)	Total (%)
SAS difficulty sleeping	Mild	1262 (18)	2310 (28)	3572 (23)
	Moderate	729 (11)	979 (12)	1708 (11)
	Severe	198 (3)	127 (1)	325 (2)
SAS appetite	Mild	1833 (26)	3439 (40)	5272 (34)
	Moderate	1269 (18)	1959 (23)	3228 (21)
	Severe	425 (6)	359 (4)	784 (5)
SAS nausea	Mild	1381 (20)	2487 (29)	3868 (25)
	Moderate	860 (12)	898 (11)	1758 (11)
	Severe	253 (4)	132 (1)	385 (2)
SAS bowels	Mild	1656 (24)	2891 (34)	4547 (29)
	Moderate	973 (14)	1113 (13)	2086 (14)
	Severe	293 (4)	210 (2)	503 (3)
SAS breathing	Mild	1283 (18)	2349 (27)	3632 (23)
	Moderate	640 (9)	609 (7)	1249 (8)
	Severe	142 (2)	67 (1)	209 (1)
SAS fatigue	Mild	1978 (28)	3416 (40)	5394 (35)
	Moderate	1893 (27)	3196 (37)	5089 (33)
	Severe	601 (9)	586 (7)	1187 (8)
SAS pain	Mild	2334 (34)	3954 (46)	6288 (40)
	Moderate	1855 (27)	1801 (21)	3656 (23)
	Severe	476 (7)	289 (3)	765 (5)
PCPSS pain	Mild	2800 (41)	4381 (51)	7181 (47)
	Moderate	1505 (22)	1499 (18)	3004 (19)
	Severe	363 (5)	221 (3)	584 (4)
PCPSS other symptoms	Mild	2986 (44)	4667 (56)	7653 (51)
	Moderate	1634 (24)	2079 (25)	3713 (25)
	Severe	339 (5)	231 (3)	570 (4)
PCPSS psychological/spiritual	Mild	3011 (44)	4784 (56)	7795 (51)
	Moderate	884 (13)	1261 (15)	2145 (14)
	Severe	158 (2)	137 (2)	295 (2)
PCPSS family/carer	Mild	2913 (43)	4620 (56)	7533 (50)
	Moderate	1130 (17)	1711 (21)	2841 (19)
	Severe	237 (3)	194 (2)	431 (3)

SAS Symptom Assessment Scale, PCPSS Palliative Care Problem Severity Score

^a^Proportion of patients who reported absent symptom-related distress have been omitted from the table

^b^SAS: A 10-point scale where a score of 0 denotes that the symptom is absent, 1–3 is mild, 4–7 is moderate and 8–10 is severe symptom distress. PCPSS: a 4-point scale, with 0 denoting that the symptom is absent, 1 is mild, 2 is moderate and 3 is severe

### Associations between distress types and symptom severity

Distress stemming from pain or appetite issues in patients with pancreatic cancer showed associations with distress due to other symptoms, as outlined in Table 5. In the inpatient setting, the strongest association was observed between the psychological/spiritual domain with patients reporting distress due to appetite (adjusted OR 1.78, 95% CI 1.66–1.90) and pain (adjusted OR 1.60, 95% CI 1.49–1.72). Similarly, in the community setting, both the pain and the psychological/spiritual domains showed the strongest association with distress related to appetite (adjusted OR 1.51, 95% CI 1.42–1.1.60 and 1.49, 95% CI 1.40–1.58, respectively) and the psychological/spiritual domain showed the strongest association with pain (adjusted OR 1.35, 95% CI 1.27–1.44).

**Table 5:** Adjusted odds ratios and 95% CIs for moderate/severe appetite-related distress and pain-related distress for levels of other symptom-related distress

Symptom		Distress due to appetite		Distress due to pain	
		Adjusted odds ratio	95% confidence interval	Adjusted odds ratio	95% confidence interval
Inpatient	SAS sleeping	1.39	1.35–1.43	1.26	1.22–1.30
	SAS nausea	1.42	1.38–1.46	1.25	1.22–1.29
	SAS bowels	1.37	1.33–1.40	1.22	1.19–1.25
	SAS breathing	1.21	1.18–1.25	1.18	1.14–1.21
	SAS fatigue	1.43	1.39–1.46	1.20	1.17–1.22
	PCPSS pain	1.37	1.30–1.45	10.37	9.25–11.64
	PCPSS other	1.60	1.51–1.69	1.30	1.23–1.38
	PCPSS psychological/spiritual	1.78	1.66–1.90	1.60	1.491–1.72
	PCPSS family	1.40	1.33–1.47	1.21	1.148–1.27
Community	SAS sleeping	1.18	1.15–1.21	1.16	1.13–1.19
	SAS nausea	1.42	1.37–1.47	1.31	1.26–1.35
	SAS bowels	1.25	1.22–1.28	1.25	1.21–1.28
	SAS breathing	1.12	1.09–1.16	1.10	1.07–1.14
	SAS fatigue	1.35	1.32–1.38	1.11	1.09–1.13
	PCPSS pain	1.51	1.42–1.60	17.92	15.90–20.20
	PCPSS other	1.33	1.27–1.39	1.19	1.13–1.25
	PCPSS psychological/spiritual	1.49	1.40–1.58	1.35	1.27–1.44
	PCPSS family	1.28	1.22–1.34	1.15	1.09–1.20

*SAS* Symptom Assessment Scale, *PCPSS* Palliative Care Problem Severity Score

### Analysis of pain- and/or appetite-related distress by AKPS

**Fig. 1:**
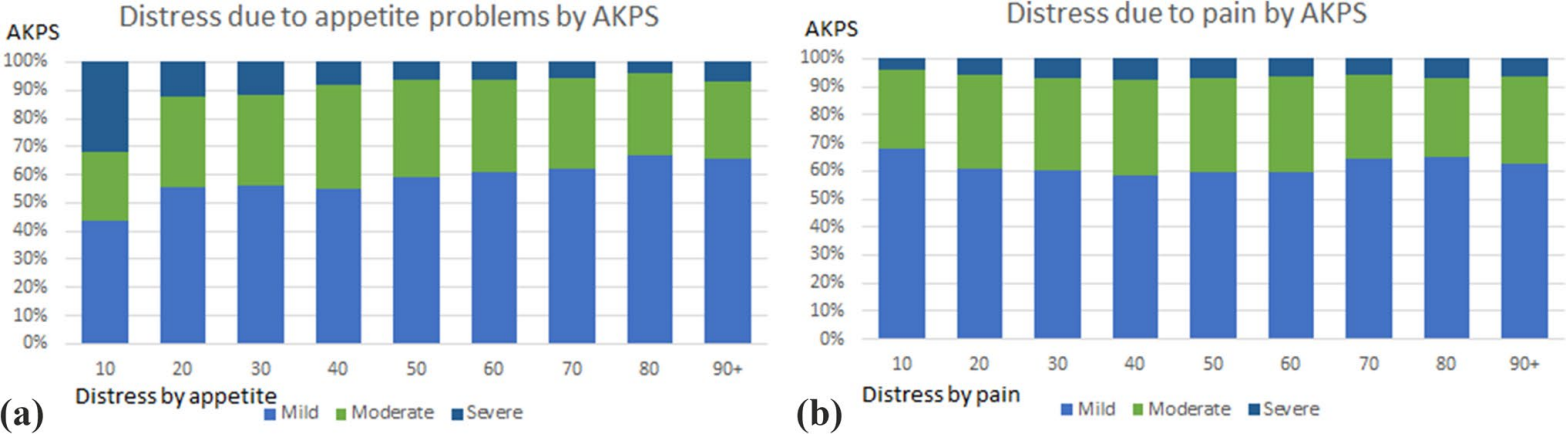
** a** Distress due to appetite problems by AKPS. **b** Distress due to pain by AKPS.

Patients reporting distress due to pain or appetite problems (constituting 60% and 69% of all patients) were further categorised based on their AKPS, as illustrated in Fig. 1a and b. While the majority of participants reported mild distress due to pain across all AKPS scores, the proportion of patients experiencing mild distress due to appetite problems most often had higher AKPS scores (80, 67% and 90 + , 66%). Notably, of those with an AKPS of 10%, 68% and 44% rated their distress due to pain and appetite problems as mild, respectively. Participants who reported moderate distress due to appetite problems were most prevalent among those with an AKPS of 40 (37%), while severe distress due to appetite problems was most frequently reported by patients with an AKPS of 10 (32%). On the other hand, the proportions of patients experiencing distress due to pain remained relatively consistent across AKPS scores for each level of distress severity (with moderate distress reported more frequently by those with a AKPS score between 20 and 60). Severe distress due to pain was experienced by only 4–7% of patients across different AKPS scores. This categorisation provides insights into the distribution of appetite- and pain-related distress across different functional states as measured by AKPS.

### Change in distress due to symptoms over time

#### Appetite

**Fig. 2:**
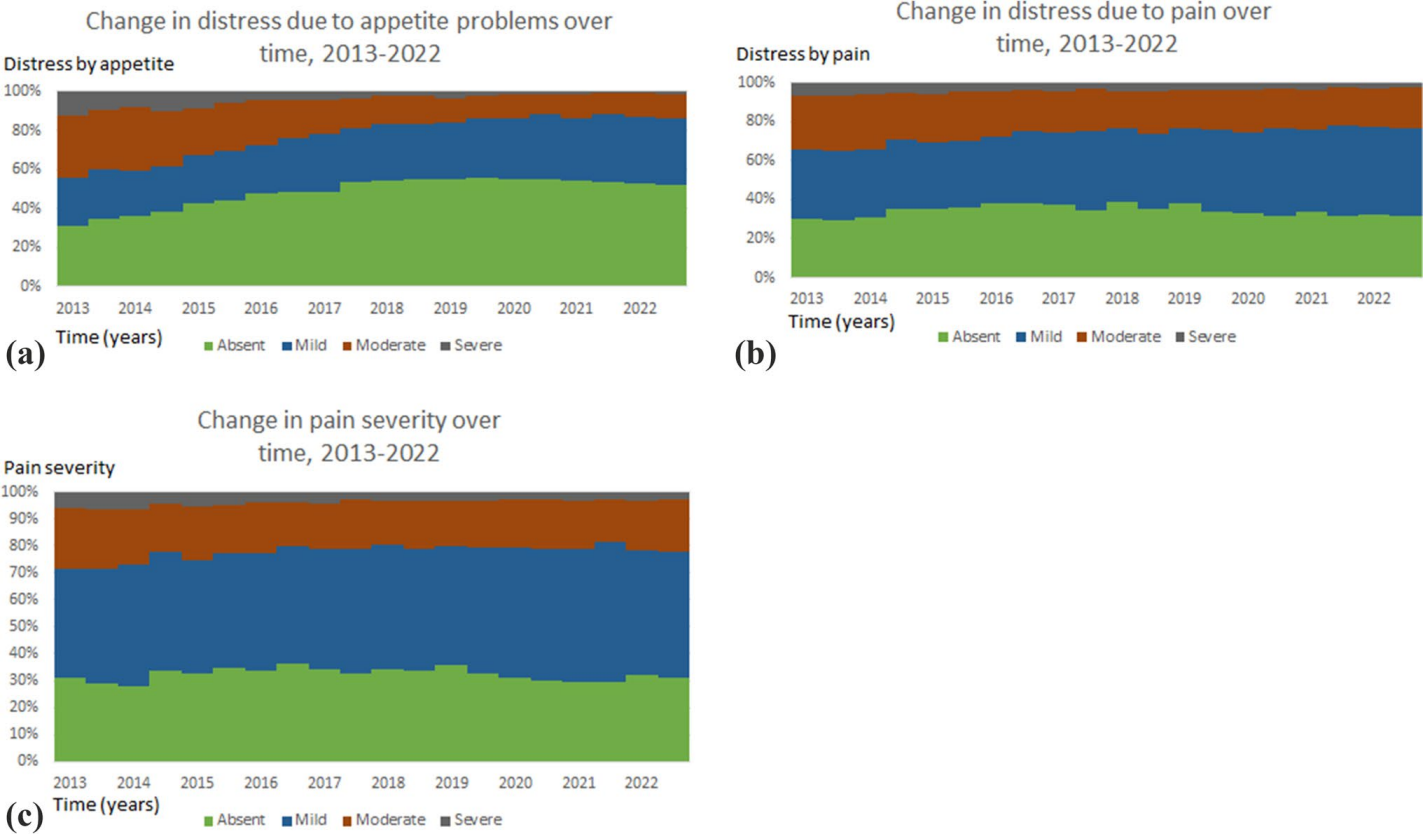
** a** Change in distress due to appetite problems over time as measured by SAS, 2013–2022. **b** Change in distress due to pain problems over time as measured by SAS 2013–2022. **c** Change in pain severity over time, as measured by the PCPSS 2013–2022

According to Fig. 2a, episodes of care reflecting absent distress due to appetite problems exhibited a notable upward trend, rising from approximately 31% in 2013 to 54% in 2017. This prevalence then remained relatively stable through the year 2022. The distribution of distress levels indicated a nuanced pattern. The proportion of patients across Australia reporting mild distress due to appetite problems increased from 25% in 2013 to 35% in 2021. Conversely, moderate distress experienced a substantial decline, dropping from 32% in 2013 to a mere 12% in 2022. Severe distress, which affected 12% of patients in 2013, saw a significant reduction to just 1% in 2022.

#### Pain

Figure 2b illustrates the changing landscape of pain-related distress levels over the period from 2013 to 2022, suggesting shifts in the prevalence and severity of pain experiences among patients and some noteworthy trends. Patients who did not report any distress due to pain showed relative consistency, comprising 30% in 2013 and 31% in 2022. An increase was observed in patients reporting mild pain, rising from 36% in 2013 to 45% in 2022. Conversely, a decrease from 27% in 2013 to 21% in 2022 in the proportion of patients reporting moderate pain was observed. Furthermore, a small proportion of patients reported severe pain, with a decline from 7% in 2013 to 3% in 2022.

### Change in pain severity over time

The additional information provided in Fig. 2c, detailing the distribution of pain levels reported by patients between 2013 and 2022, further contributes to the understanding of pain-related distress trends. During this period, patients reporting mild pain showed a slightly increasing range of 41–52%, indicating some variability over the years. Conversely, those reporting moderate pain demonstrated a decreasing ranged from 22 to 16%, while those reporting severe pain decreased from 6 to 3% between 2013 and 2022. These fluctuations in ranges imply ongoing variations in the prevalence of pain experiences among patients over the studied timeframe.

## Discussion

Our data spanning 2013 to 2022 is one of the largest cohorts providing insights into the demographic profile and care settings for patients with pancreatic cancer at presentation to palliative care services in Australia. Our findings showed a high prevalence of distress related to pain and appetite at the initial episode of care, with over half reporting distress in these areas. This suggests the need for both more effective symptom management strategies and early engagement of palliative care, ideally with referrals at the time of diagnosis or shortly thereafter. This in turn can facilitate timely interventions to relieve symptom burden and its associated distress. Especially considering that, at most, 86% of patients with pancreatic cancer experience at least one moderate severe symptom following systemic treatment [22].

Patients with distress due to pain or appetite were typically older, had lower functional status and faced a substantial burden of other symptoms and psychosocial needs. Most patients also initiated their palliative care within the community setting, underscoring the importance of accessible and community-centred healthcare services for this population group. Sex differences were negligible in any parameter. An inverse relationship between age and distress from pain was observed although the basis for this difference is not apparent from the data in this series. However, this trend has been observed in existing literature [12]. Distress due to appetite issues was prevalent across all age groups indicating the significance of this symptom in patients with pancreatic cancer, irrespective of age.

Distress was evident across all levels of severity for appetite issues and pain, although the majority of patients rated these symptoms as mild. This trend mirrors findings among patients with pancreatic cancer in Victoria, Australia, where only 19% reported severe pain scores (≥ 4) on PCOC SAS at first episode [12]. Patients reporting distress related to symptoms other than pain and appetite also experienced moderate to severe levels of pain and appetite symptoms, suggesting the significant impact of symptom distress on patient’s quality-of-life. It has been recognised that enduring symptoms lead to distress by disrupting daily activities and diminishing quality-of-life [23]. In addition, the strong associations between distress related to pain and appetite with distress in the psychological/spiritual domain suggest the interconnectedness of physical symptoms with psychological well-being, highlighting the need for integrated palliative care approaches. Typically, addressing spiritual concerns has not been a primary focus for healthcare professionals delivering palliative care despite research demonstrating the significance of spiritual care for patients at the end-of-life [24].

Nausea also emerged as a significant symptom contributing to distress in patients with pancreatic cancer. Nausea itself can significantly reduce food intake, compounding on existing anorexia [25]. Furthermore, our findings indicated high prevalence of family/carer issues associated with pain and appetite-related distress experienced by their loved ones. Caregivers are often affected by the patient’s pain, inability to eat, evident weight loss and social isolation through reduced interaction with family. These challenges can serve as prominent reminders of the gravity of the situation and often contribute to tension between patient and caregiver [26]. Collectively, the co-occurrence of distress related to multiple symptoms suggests the multifaceted nature of symptom burden in pancreatic cancer patients under palliative care and their caregivers, underlying the need for comprehensive symptom management strategies that address the interconnected domains of pain, psychological/spiritual well-being, family/carer issues and other symptoms.

Our study also demonstrated dynamic changes in the prevalence and severity of distress related to appetite and pain at the commencement of first palliative care episode over time. Notable shifts in distress levels, particularly the reduction in severe distress over the years is unsurprising as previous research has shown a similar trend among patients with pancreatic cancer with a decreasing likelihood of experiencing high distress related to pain over time [12]. This may indicate potential improvements over the years in symptom management practices or changes in patient care approaches, reflecting potential advancements in timing of access, engagement/delivery and support to palliative care services for patients with pancreatic cancer. Our findings underscore the importance of continuous monitoring to understand the factors contributing to these variations, such as changes in treatment approaches, patient demographics or healthcare policies and how healthcare interventions may have influenced these trends.

### Strengths and limitations of the study

The study’s longitudinal design spanning from 2013 to 2022 in addition to the inclusion of data from a very large and heterogeneous cohort of patients, both allow for the examination of trends and changes over an extended period as well as enhances generalisability of the findings, providing a comprehensive understanding of the dynamics of distress in patients with pancreatic cancer. That said, PCOC is a voluntary service and therefore, does not capture all palliative care presentations. In addition, patients who present to multiple services cannot be uniquely identified and therefore, may have been counted more than once. While the reliance on patient-reported data provides a more patient-centred understanding of symptoms of distress due to appetite problems and pain, information on the source of the PCOC SAS score is not routinely collected by PCOC. It is important to note that proxy ratings, which may differ from patient ratings, are a possibility. Furthermore, given the observational nature of the data, it is crucial to emphasise that no causal relationships can be inferred from the results. PCOC specifically does not capture details of care plans; instead, it focuses solely on measuring care outcomes, such as if appetite/pain distress increased or not. Consequently, the data may not provide insights into the specific changes in care responsible for variations. In addition, lack of data collected regarding date of diagnosis does not allow for interrogation of timing of referral to palliative care, nor the therapies that may have been used prior to referral. Indeed, the most proximate data available are when patients were referred to a palliative care service. Any changes in symptom control can only reflect a before-and-after relationship, with no ability to ascribe causality. Finally, the definition or criteria used to classify services as specialist palliative care may vary, and this lack of standardised criteria could introduce ambiguity in interpreting and comparing results. In this setting, a potential limitation to our study is the small proportion of services contributing data, which may have been perceived as specialist palliative care services. Given the limitations outlined, additional rigorous studies are warranted to validate the reported observations and establish causal relationships within the context of palliative care.

## Conclusion

Our analysis of a large data set on episodes of palliative care for people with pancreatic cancer in Australia reveals the complex interplay between demographic factors, clinical characteristics, symptom burden and distress levels from pain and appetite. Furthermore, the study highlights the importance of considering the setting in which distress manifests, with distinct associations observed in inpatient and community settings. Psychological and spiritual issues emerged as a significant contributor to distress related to appetite, particularly in the inpatient setting, highlighting the need for holistic care approaches that address both physical and psychological dimensions. Pain severity consistently emerged as a major factor associated with distress, indicating the crucial role of patient-centred care with a focus on tailoring effective pain management interventions based on the severity of reported pain and addressing individual patient needs to improving overall patient well-being. These findings emphasise the multifaceted nature of distress experienced by people with pancreatic cancer. As healthcare professionals strive to enhance the quality of care for these individuals, a comprehensive understanding of the interplay between various factors influencing distress becomes paramount. Ultimately, these insights can guide the development of targeted strategies addressing specific symptoms and settings to improve the overall quality-of-life for individuals facing pancreatic cancer within the Australian healthcare system.

## Data Availability

No datasets were generated or analysed during the current study.
